# Accuracy of abdominal ultrasound for the diagnosis of pneumoperitoneum in patients with acute abdominal pain: a pilot study

**DOI:** 10.1186/s13089-015-0032-6

**Published:** 2015-10-06

**Authors:** Peiman Nazerian, Camilla Tozzetti, Simone Vanni, Maurizio Bartolucci, Simona Gualtieri, Federica Trausi, Marco Vittorini, Elisabetta Catini, Gian Alfonso Cibinel, Stefano Grifoni

**Affiliations:** Department of Emergency Medicine, Careggi University Hospital, largo Brambilla 3, 50134 Florence, Italy; Radiology Department, Careggi University Hospital, Florence, Italy; Department of Emergency Medicine, Pinerolo Hospital, Turin, Italy

**Keywords:** Pneumoperitoneum diagnosis, Abdominal ultrasound, Abdominal radiography, Abdominal pain, Hollow organ perforation diagnosis

## Abstract

**Background:**

Pneumoperitoneum is a rare cause of abdominal pain characterized by a high mortality. Ultrasonography (US) can detect free intraperitoneal air; however, its accuracy remains unclear. The aims of this pilot study were to define the diagnostic performance and the reliability of abdominal US for the diagnosis of pneumoperitoneum.

**Methods:**

This was a prospective observational study. Four senior and two junior physicians were shown, in an unpaired randomized order, abdominal US videos from 11 patients with and 11 patients without pneumoperitoneum. Abdominal US videos were obtained from consecutive patients presenting to ED complaining abdominal pain with the diagnosis of pneumoperitoneum established by CT. Abdominal US was performed according to a standardized protocol that included the following scans: epigastrium, right and left hypochondrium, umbilical area and right hypochondrium with the patient lying on the left flank. We evaluated accuracy, intra- and inter-observer agreement of abdominal US when reviewed by senior physicians. Furthermore, we compared the accuracy of a “2 scan-fast exam” (epigastrium and right hypochondrium) vs the full US examination and the accuracy of physicians expert in US vs nonexpert ones. Finally, accuracy of US was compared with abdominal radiography in patients with available images.

**Results:**

Considering senior revision, accuracy of abdominal US was 88.6 % (95 % CI 79.4-92.4 %) with a sensitivity of 95.5 % (95 % CI 86.3–99.2 %) and a specificity of 81.8 % (95 % CI 72.6–85.5 %). Inter- and intra-observer agreement (k) were 0.64 and 0.95, respectively. Accuracy of a “2 scan-fast exam” (87.5 %, 95 % CI 77.9–92.4 %) was similar to global exam. Sensitivity of abdominal radiography (72.2 %, 95 % CI 54.8–85.7 %) was lower than that of abdominal US, while specificity (92.5 %, 95 % CI 79.5–98.3 %) was higher. Accuracy (68.2 %, 95 % CI 51.4–80.9 %) of junior reviewers evaluating US was lower than senior reviewers.

**Conclusions:**

Senior physicians can recognize US signs of pneumoperitoneum with a good accuracy and reliability; sensitivity of US could be superior to abdominal radiography and a 2 fast-scan exam seems as accurate as full abdominal examination. US could be a useful bedside screening test for pneumoperitoneum.

Trial registry ClinicalTrials.gov; No.: NCT02004925; URL: http://www.clinicaltrials.gov

**Electronic supplementary material:**

The online version of this article (doi:10.1186/s13089-015-0032-6) contains supplementary material, which is available to authorized users.

## Background

Pneumoperitoneum most commonly results from a perforated hollow viscus. It is a rare cause of acute abdominal pain representing less than 1 % of presentation to emergency department (ED) and its prompt diagnosis is paramount due to its high mortality.

Clinical signs and symptoms have low diagnostic accuracy and abdominal radiography is positive in 55–85 % of cases [1–5]. Computed tomography (CT) is considered the “gold standard” for the recognition of free intraperitoneal air; however, is not a cost-effective option as a screening test for patients with acute abdominal pain, exposes to significant radiations, is not always available in every centers and requires patients to be transferred for examination [6, 7].

**Fig. 1:**
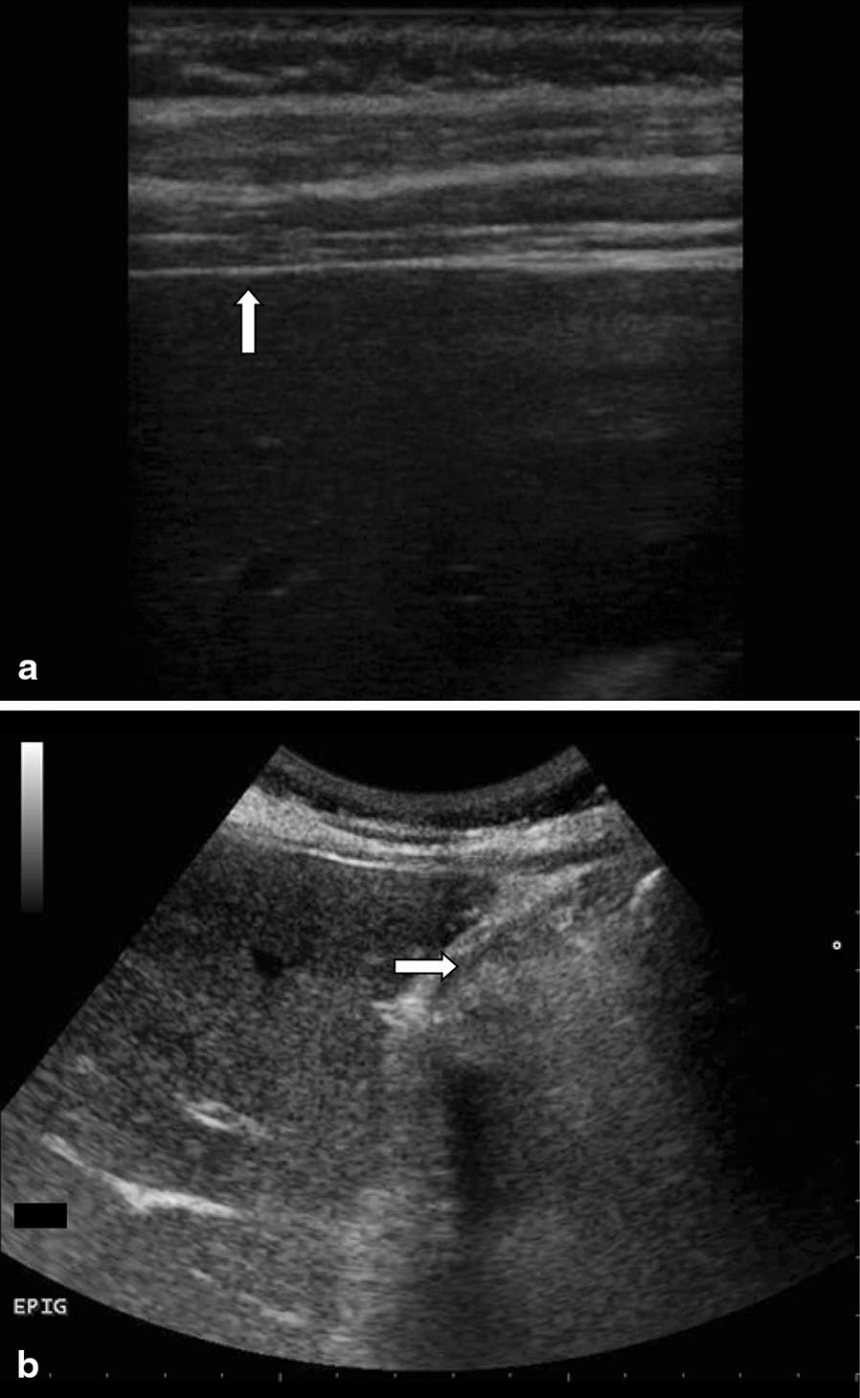
**a** Normal peritoneal stripe (*white arrow*) in a patient without pneumoperitoneum in right hypochondrium scan with linear probe. **b** Normal air within the lumen of the gastrointestinal tract, recognizable by its association with bowel (*white arrow*) in a patient without pneumoperitoneum in right hypochondrium scan with convex probe

**Fig. 2:**
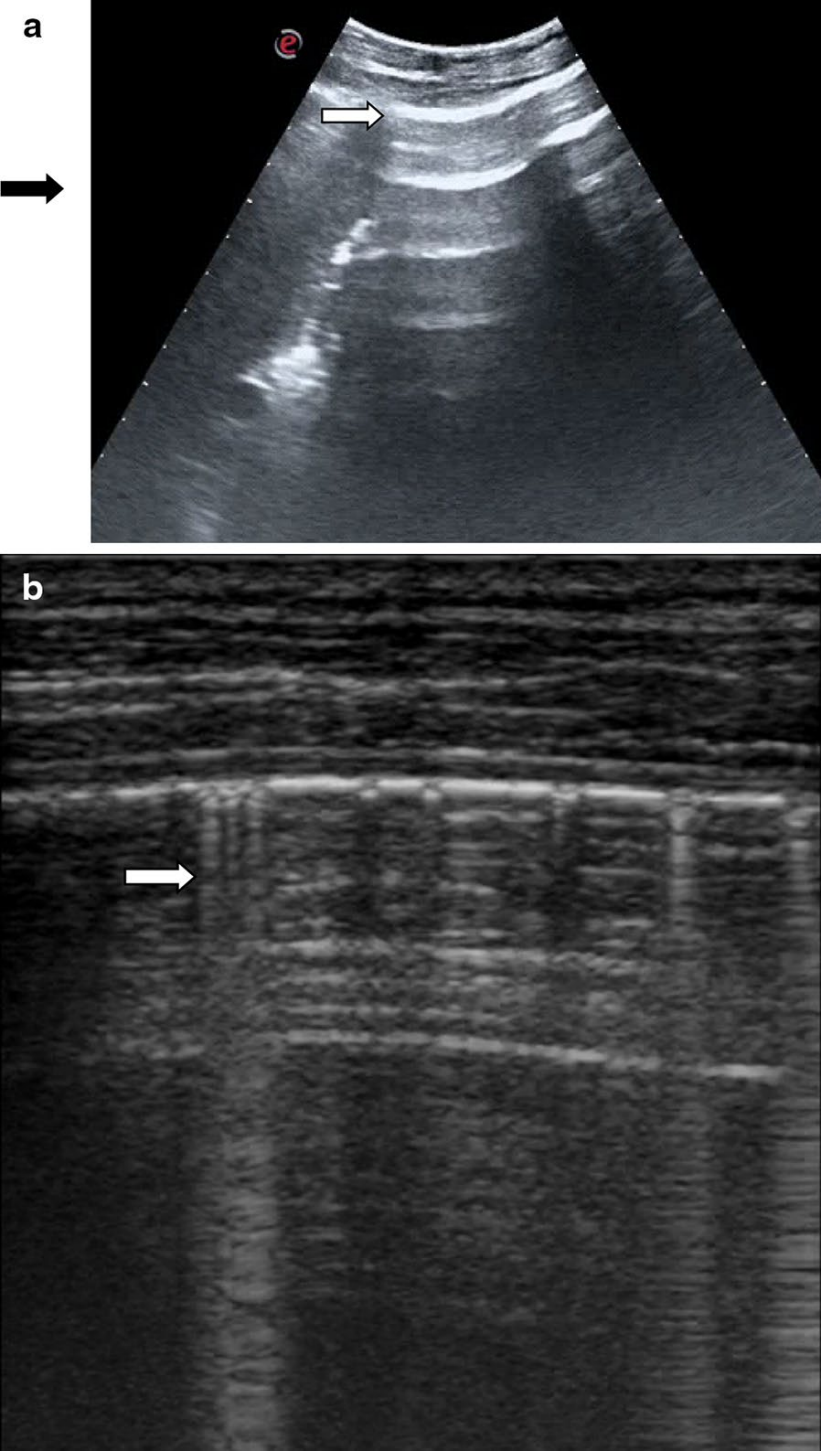
**a** Enhancement of peritoneal stripe (*white arrow*) and reverberation artifacts (*black arrow*) detected in the right hypochondrium scan with convex probe in a patient with pneumoperitoneum. **b** Reverberation with a ring down artifact “comet tails” (*white arrow*) starting from peritoneum detected in the right hypochondrium scan with linear probe in a patient with pneumoperitoneum

The use of bedside ultrasonography (US) to diagnose abdominal aneurysm, biliary colic/cholecystitis, hydronephrosis and free intra-abdominal fluid in patients presenting to the ED with abdominal pain is well established [8, 9]. Previous studies evaluated diagnostic performance of abdominal US to detect free intraperitoneal air demonstrating that US has a superior sensitivity to diagnose pneumoperitoneum compared with abdominal radiography with the most important sonographic findings including the enhancement of peritoneal stripe and reverberation with a ring down artifact starting from peritoneum [10–20]. The normal peritoneal stripe is identified by US as a single or double echogenic line posterior to the anterior abdominal wall (Fig. 1a) and normal air within the lumen of the gastrointestinal tract is recognizable by its association with bowel and moves with peristalsis (Fig. 1b). In pneumoperitoneum, free abdominal air produces a sonographic appearance of linear enhancement of peritoneal stripe with reverberation artifacts (Fig. 2a; Additional file 1), and the high differences in acoustic impedance between closely opposed air and fluid produce ring down artifacts starting from peritoneal line (Fig. 2b; Additional file 2).

The accuracy of US in detecting free intraperitoneal air in the acute setting remains unclear and the best US protocol to be performed in ED to screen patients with suspected pneumoperitoneum is still unknown, furthermore inter- and intra-observer agreement among different physicians and accuracy of physicians with different level of expertise are never been evaluated in previous studies.

The main aims of this pilot study were to define the accuracy and reliability of abdominal US for the diagnosis of pneumoperitoneum. Furthermore, we evaluated the accuracy of the linear vs convex probe and the accuracy of a “2 scan-fast exam” (epigastrium and right hypochondrium scans with convex probe) vs a full abdominal examination and we also compared the accuracy of physicians expert in US with nonexpert ones in recognizing US signs of pneumoperitoneum.

## Methods

### Study design and setting

This was a prospective single-center blinded observational study. The local ethic committee approved the study (protocol number 2014/9735) and the study was registered in ClinicalTrials.gov (NCT02004925).

### Study protocol

Four physicians with at least 3 years of experience in abdominal US but without previous experience in the evaluation of US signs of pneumoperitoneum (senior reviewers) and two second year internal medicine residents with no previous experience in abdominal US (junior reviewers) were given the same 30-min tutorial on how to recognize pneumoperitoneum on both US and radiography. Senior reviewers were one attending emergency physician, one attending radiologist, one fifth year resident in emergency-internal medicine and one fourth year resident in radiology with 8, 15, 3 and 3 years of experience in abdominal US, respectively. All reviewers were shown abdominal US videos from 11 patients with (cases) and 11 patients without pneumoperitoneum (controls) and abdominal radiography of the same patients when available.

Abdominal US videos were obtained from consecutive patients aged >18 years, presenting to an ED of an adult tertiary university hospital with an annual census of 130,000 visits, staffed with 24 emergency physicians. Consenting patients were prospectively included in the study, if the following criteria were satisfied: (1) cases—patients with acute abdominal pain and pneumoperitoneum diagnosed by CT of the abdomen in ED; (2) controls—adult patients with severe acute abdominal pain (numeric rating scale >7) associated with signs of acute abdomen or shock/hypotension (systolic arterial blood pressure <90 mmHg) with diagnosis other than pneumoperitoneum by CT. CT of the abdomen was performed with a Somatom Definition AS128 (Siemens, Erlangen, Germany) in the ED. Trauma patients were excluded from the study.

Abdominal US was performed after CT by one of two emergency physicians sonographers with 2 years of experience in abdominal US. The following machines equipped with a 3.5–5 MHz convex and 4–8 MHz linear probe were used: MyLab30 Gold, MyLab Alpha (Esaote, Genova, Italy) and HD7 (Philips, Amsterdam, Holland). All patients underwent a standardized US protocol that included the following scans: epigastrium, right hypochondrium, left hypochondrium, umbilical area and right hypochondrium with the patient lying for at least 2 min on the left flank. The scans were performed both with linear and convex probes starting randomly with one of the two probes; the probe was gently held on patients’ skin without pressing with the aim of avoiding the compression of intraperitoneal air and the depth was adjusted to have the peritoneum line in the half upper part of the screen. All scans were recorded in a video of 5 s each.

Abdominal radiography was performed when requested by the treating physician with a Practix 300 or a Bucky diagnost (Philips Medical Systems, Hamburg Germany) in the upright position and when patients were not able to stand-up in left lateral decubitus whenever possible.

Videos of abdominal US and radiograph images were digitally stored and were shown to reviewers separately in an unpaired randomized order using the same personal computers used for interpretation of radiological investigations in the ED’s daily practice.

In particular, the US videos corresponding to scans of different anatomical area of the abdomen of the same patient were shown to the reviewers for 5 s each in a random order. The reviewers, after evaluating each video maximum twice, fulfilled a standardized form (Additional file 3) signing for each video if the following signs of pneumoperitoneum were present or absent: enhancement of the peritoneum stripe (Fig. 2a; Additional file 1) and reverberation with a ring down artifact or comet tails starting from peritoneum (Fig. 2b; Additional file 2). If one of the signs of pneumoperitoneum was present in at least one scan, the patient was considered to have an ultrasonographic diagnosis of pneumoperitoneum. Senior reviewers reevaluated the US videos after 2 months with the aim of calculating intra-observer agreement. Images of abdomen radiography were examined by reviewers for 1 min for each patient to establish the presence of pneumoperitoneum. Reviewers were blinded to all patients’ medical information.

### Data analysis

Dichotomous data are expressed as proportions and continuous data are expressed as mean ± standard deviation (SD). Fisher’s exact test was used for the comparison of dichotomous data, and the unpaired Student’s *t* test was used to compare normally distributed data. The diagnostic performance of senior reviewers in detecting ultrasonographic signs of pneumoperitoneum was calculated by computing accuracy, sensitivity, specificity, negative and positive predictive values and negative and positive likelihood ratios with their 95 % confident interval (CI), using at least one positive scan for pneumoperitoneum signs either with convex or linear probe. The diagnostic performances of linear and convex probe and of a 2 scan-fast exam consisting of right hypochondrium and epigastrium were calculated in the same way. The diagnostic performance of senior reviewers in detecting pneumoperitoneum after reviewing abdominal radiography in patients with available radiography and the diagnostic performance of junior reviewers in detecting US sign and radiological signs of pneumoperitoneum were also calculated. The extended McNemar and the McNemar tests were used to evaluate if there was a significant difference in the accuracy, sensitivities and specificities. Inter-observer agreement among senior reviewers was assessed using the Fleiss’ kappa (k) while intra-observer agreement was calculated using Cohen’s kappa (k). Analysis was performed with the SPSS statistical package (version 17.0, SPSS Inc., Chicago, Illinois).

## Results

The 11 cases with pneumoperitoneum and the 11 controls without pneumoperitoneum were recruited from 1st of March 2014 to 29th May 2014. During this period 2502 patients presented to the ED complaining abdominal pain; abdominal US, abdominal radiography and CT of the abdomen were performed in 1488 (59.4 %), 1116 (46.4 %) and 132 (5.3 %) patients, respectively. Fifteen patients (0.6 %) were diagnosed with pneumoperitoneum by CT. Of these, 3 patients were excluded from the study because patients were immediately transferred to the surgical theater and the EP sonographer could not perform the abdominal US in the ED, and one case was excluded because of denied consent.

Table 1 reports the clinical characteristics of enrolled patients. Nine (81 %) patients with a final diagnosis of pneumoperitoneum, underwent surgery, 1 received conservative treatment and 1 died in ED. Among controls, 6 (54 %) patients underwent surgery. In all cases and controls, intra-operative findings confirmed CT diagnosis about presence or absence of pneumoperitoneum. Pneumoperitoneum was due to gastric or duodenal perforation of peptic ulcer in 3 cases, to perforation of colic diverticulitis in 3 cases, to small bowel perforation secondary to pancreatic neoplasia in one case, to colon perforation in 4 cases. Regarding control patients the final diagnosis were: 3 bowel occlusion, 4 peritonitis in 1 case due to appendicitis, in 2 to diverticulitis, and in 1 to cholecystitis, 1 pancreatitis, 2 ischemic bowel disease, 1 hemoperitoneum secondary to ovarian cystic rupture.

**Table 1:** Clinical characteristics in patients with and without pneumoperitoneum

	Pneumoperitoneum cases = 11	No. pneumoperitoneum controls = 11	*p*
Female gender	5 (45 %)	4 (36 %)	0.5
Age (years)	68 ± 17.1	66.4 ± 25.7	0.87
Anamnestic features			
Previous abdominal surgery	2 (18 %)	5 (45 %)	0.15
Active neoplasia	3 (27 %)	1 (9 %)	0.29
Symptoms at presentation			
Pain scale (NRS)	7.4 ± 1.6	7.6 ± 1.3	0.77
Vomit	2 (18 %)	4 (36 %)	0.32
Physical findings			
Signs of peritonism	6 (54 %)	5 (45 %)	0.5
Abdominal distension	9 (81 %)	8 (72 %)	0.5
Shock/hypotension (SBP <90 mmHg)	2 (18 %)	1 (9 %)	0.5

Values are reported as mean ± standard deviation for continuous variables or as absolute number and percent value (in brackets). *p* significant if <0.05

*NRS* numeric rating scale, *SBP* systolic blood pressure

Considering senior revision, accuracy of abdominal US (one scan positive among those obtained with convex or linear probe) was 88.6 % (95 % CI 79.4–92.4 %) with a sensitivity of 95.5 % (95 % CI 86.3–99.2) and a specificity of 81.8 % (95 % CI 72.6–85.5 %). Table 2 reports the diagnostic performance of abdominal US based on senior revision.

**Table 2:** Diagnostic performance of abdominal ultrasonography and abdominal radiography for the diagnosis of pneumoperitoneum based on senior revision

	Sensitivity (95 % CI)	Specificity (95 % CI)	PPV (95 % CI)	NPV (95 % CI)	+LR (95 % CI)	−LR (95 % CI)
US exam^a^	95.5 (86.3–99.2)	81.8 (72.6–85.5)	84.0 (75.9–87.3)	94.7 (84.1–99.0)	5.25 (3.15–6.85)	0.05 (0.01–0.18)
2 scan-fast US^b^	93.2 (83.6–98.1)	81.8 (72.3–86.7)	83.7 (75.1–88.1)	92.3 (81.5–97.8)	5.12 (3.01–7.38)	0.08 (0.02–0.22)
X-ray^c^	72.2 (54.8–85.7)	92.5 (79.5–98.3)	89.6 (72.6–97.6)	78.7 (64.3–89.2)	9.63 (3.18–29.13)	0.30 (0.18–0.51)

*US* ultrasound, *PPV* positive predictive value, *NPV* negative predictive value, +*LR* positive likelihood ratio, −*LR* negative likelihood ratio, *95* *% CI* confidence interval

^a^One scan positive among those obtained with convex or linear probe

^b^One scan positive among right hypochondrium and epigastrium scans with convex probe

^c^Considering 19 patients with available abdominal radiography

**Fig. 3:**
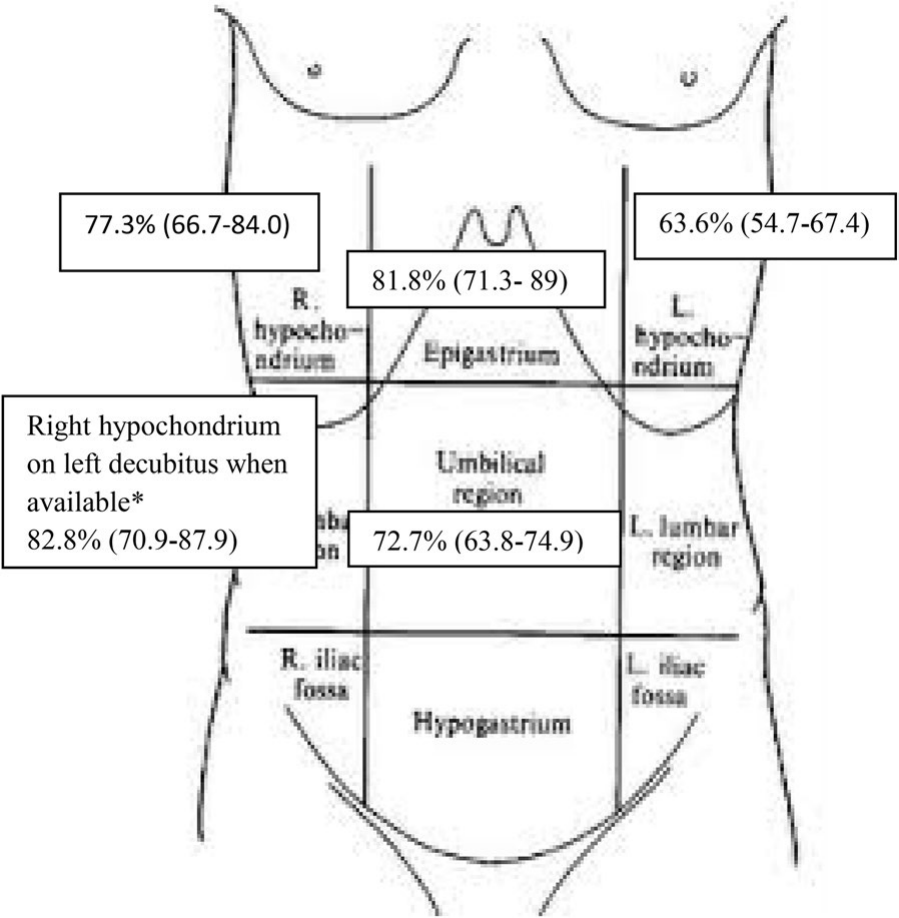
Accuracy of each single convex scan according to seniors’ revision. *Asterisk* 8 patients with pneumoperitoneum and 9 patients without

**Fig. 4:**
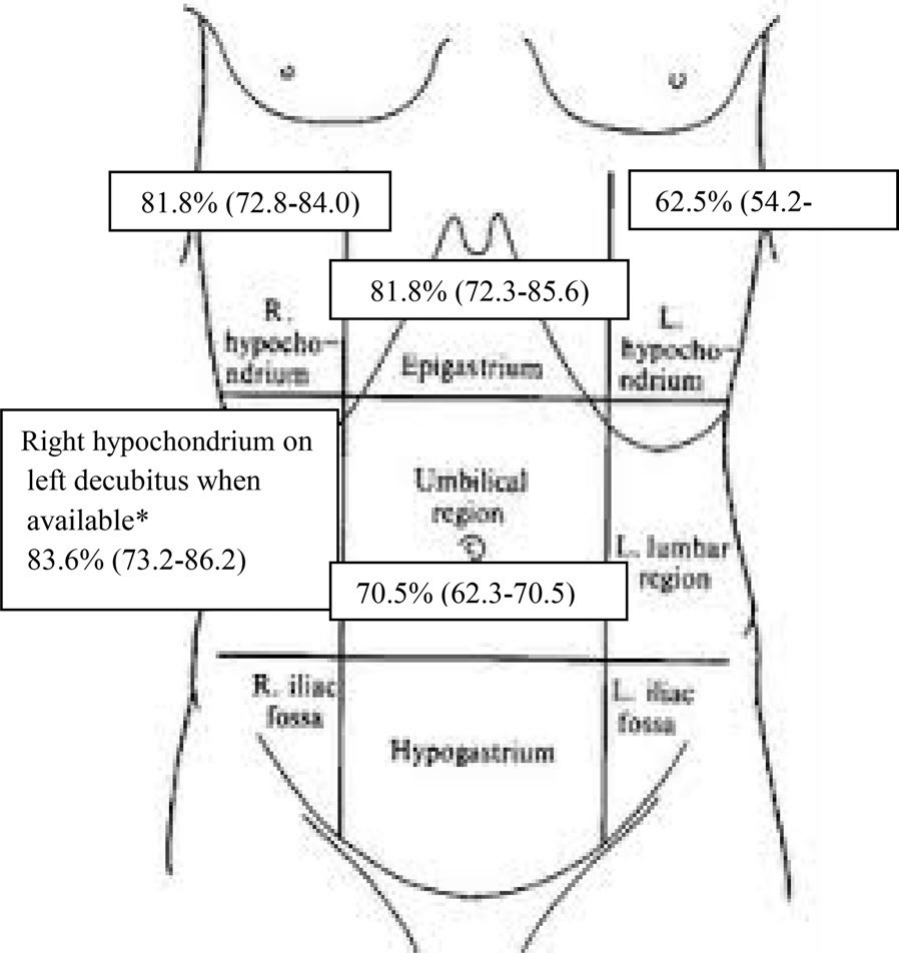
Accuracy of each single linear scan according to seniors’ revision. *Asterisk* 8 patients with pneumoperitoneum and 9 patients without

Inter-observer agreement between the four senior reviewers was 0.64 while intra-observer agreement was 0.95. Accuracy of convex and linear probes was 85.2 % (95 % CI 75.1–91.7 %) and 89.8 % (95 % CI 80.7–93.5 %), respectively. Sensitivities of convex and linear probe were similar (88.6 %, 95 % CI 78.5–95.1 % vs 84.1 %, 95 % CI 75–87.8 %, *p* = 0.29), while specificity of convex was lower than linear probe (81.8 %, 95 % 71.7–88.3 % vs 95.5 %, 95 % CI 86.4–99.2 % *p* = 0.01). Accuracy of a “2 scan-fast exam” performed with convex probe (87.5 %, 95 % CI 77.9–92.4 %) was similar to global exam (*p* = 0.37) (Table 2). Figures 3 and 4 report accuracy and 95 % CI of abdominal US performed with convex and linear probes in different abdominal areas.

Abdominal radiography was performed in 19 patients (9 cases and 10 controls). In 8 patients the exam was performed in the upright position, in 6 patients in the left lateral decubitus and in 5 patients only in the supine position. According to senior revision, sensitivity of abdominal radiography (72.2 %, 95 % CI 54.8–85.7 %) was lower than of abdominal US (86 %, 95 % CI 74–94 %*)* (*p* = 0.001), while specificity of radiography (92.5 %, 95 % CI 79.5–98.3 %) was higher than of US (80 %, 95 % CI 69–87 %) but without reaching statistical significance (*p* = 0.29) (Table 2).

Accuracy (68.2 %, 95 % CI 51.4–80.9 %), sensitivity (77.3 %, 95 % CI 60.5–90 %) and specificity (59.1 %, 95 % CI 42.3–71.8 %) for pneumoperitoneum diagnosis based on junior revision of abdominal US were lower when compared with senior reviewers (p < 0.05). Accuracy, sensitivity and specificity of junior reviewers for detecting pneumoperitoneum based on abdominal radiography revision were 76 % (95 % CI 55–87 %), 67 % (95 % CI 41–87 %) and 85 % (95 % CI 62–97 %), respectively.

## Discussion

Hollow organ perforation is a surgical life-threatening emergency that presents with acute abdominal pain. US is often used as an initial diagnostic tool to evaluate patients with acute abdominal pain in the ED and incorporating the assessment of US pneumoperitoneum signs in the standard abdominal US protocol may provide faster and more accurate diagnosis at the bedside. Several previous studies, in accordance with this study, showed that abdominal US has a good accuracy for the diagnosis of pneumoperitoneum with a sensitivity ranging from 86 to 94 % and a specificity ranging from 53 to 100 % [10–18]. It is important to bear in mind that for a life-threatening condition such as pneumoperitoneum, US can be used as a screening test but it cannot be used as a stand-alone test to rule out pneumoperitoneum in high clinical suspected cases, nor can be used to rule in pneumoperitonuem and to send patients for surgery without a confirmative test such as CT.

In our observational study we defined prior sonographic signs of pneumoperitoneum: enhancement of peritoneal stripe and reverberation with a ring down artifact starting from peritoneum, the most validated signs in previous studies [10–19]. For the first time, we demonstrated that intra- and inter-observer agreement among physicians with different background is good when considering the interpretation of these two US signs.

Both linear and convex probes were accurate for detecting US signs of pneumoperitoneum even if linear probe showed superior specificity due to its high near-field resolution compared to convex probe. More often pneumoperitoneum is diagnosed on US by detection of air between the anterior abdominal wall and the liver because these are the most non-dependent parts of the abdominal cavity where abdominal gas can accumulate between the parietal peritoneum and the liver and is therefore more easily detected. One purpose of our study was to investigate the accuracy of a “2 scan-fast exam” (epigastrium and right hypochondrium scans with convex probe) and we found a similar accuracy among a “2 scan-fast exam” (87 %) compared with a five scans abdominal examination (89 %). Chen et al., using right hypochondrium and epigastrium scans with convex probe found an accuracy of 88 % similar to our “2 scan-fast exam”[10, 11]. We can argue that if a sonographer is familiar with the two mentioned sonographic signs of pneumoperitoneum, while scanning epigastrium and right hypochondrium with convex probe (scans that are normally performed in patients with acute abdominal pain) can screen patients for pneumoperitoneum without any delay in the examination. Sonographer has only to remind to not press deeply the abdominal wall with the probe while evaluating the patient for presence or absence of pneumoperitoneum. In doubtful cases other abdominal scans can be added and also the convex probe can be changed to linear.

Comparing abdominal US with abdominal radiography, we showed that radiographic signs of pneumoperitoneum are detected with less sensitivity than US signs (72 % vs 86 %) while specificity of radiography was higher even if statistical significance was not reached (92.5 % vs 80 %), showing that US and radiography can be used as complementary tests to increase accuracy.

Finally, diagnostic accuracy of junior reviewers (68 %) without any experience in US was lower than senior reviewers (89 %), although senior reviewers did not have previous experience in evaluating US signs of pneumoperitoneum. This demonstrated that physicians once experienced in ultrasonography can become proficient in recognizing US signs of pneumoperitonuem with a very short training, while physicians with no ultrasonographic experience need further training.

### Limitations

Interpretation of our results is limited by the small sample size and needs elucidation through further studies. As the purpose of this study was to assess image interpretation, reviewers were not provided with any clinical data; hence, the diagnostic performance could be different if these information are known. Furthermore, interpreting a video with the aim of detecting the presence of pneumoperitoneum could not be the same as when performing the US exam in “real time” at the bedside. Moreover, other signs of pneumoperitoneum like “scissor sign” or the presence of fluid in peritoneal cavity, consequence of intestinal perforation, were not directly addressed in this study. When considered together, these signs could enhance the accuracy of US in the diagnosis of intestinal perforation. Finally, when comparing US with abdominal radiography, we have to consider that half of the senior reviewers were non-radiologists and this could have led to a reduced accuracy of radiography.

## Conclusions

US can be a useful bedside screening test for pneumoperitoneum in patients with acute abdominal pain. This pilot study showed that physicians with previous US experience after a short training could recognize US signs of pneumoperitoneum with a good accuracy and inter- and intra-observer agreement. Furthermore, this pilot study suggests that sensitivity of US could be superior to abdominal radiography and that a 2 fast-scan exam could be as accurate as full abdominal examination; however, more studies are needed to confirm these preliminary findings in a larger ED population.

## Additional files

10.1186/s13089-015-0032-6 Enhancement of peritoneal stripe and reverberation artifacts detected in the right hypochondrium scan with convex probe in a patient with pneumoperitoneum.
10.1186/s13089-015-0032-6 Reverberation artifacts starting from peritoneum detected in the right hypochondrium scan with linear probe in a patient with pneumoperitoneum.
10.1186/s13089-015-0032-6 Standardized form.

## Abbreviations

ED: Emergency department
CT: Computed tomography
US: Ultrasonography
SD: Standard deviation
CI: Confident interval
K: Kappa
