# Supplementary material for: Accuracy of abdominal ultrasound for the diagnosis of pneumoperitoneum in patients with acute abdominal pain: a pilot study
Source: Crit Ultrasound J. 2015 Oct 6;7:15. doi: 10.1186/s13089-015-0032-6 (PMC4595408; doi:10.1186/s13089-015-0032-6)
Supplement: Supplementary file 3 — 10.1186/s13089-015-0032-6 Standardized form. [file 13089_2015_32_MOESM3_ESM.pdf]

Reviewer name	
Patient number	

CONVEX Probe

**Abdominal scan**	**Pneumoperitoneum US sign present***	**Pneumoperitoneum US sign absent**
EPIGASTRIUM		
RIGHT HYPOCHONDRIUM		
LEFT HYPOCHONDRIUM		
RIGHT HYPOCHONDRIUM ON THE LEFT FLANK		
UMBELICAL		
Comments		
* Enhancement of peritoneal stripe plus ring down artifact or comet tails starting from peritoneum		

LINEAR Probe

**Adominal scan**	**Pneumoperitoneum US sign present***	**Pneumoperitoneum US sign absent**
EPIGASTRIUM		
RIGHT HYPOCHONDRIUM		
LEFT HYPOCHONDRIUM		
UMBELICAL		
RIGHT HYPOCHONDRIUM ON THE LEFT FLANK		
Comments		
* Enhancement of peritoneal stripe plus ring down artifact or comet tails starting from peritoneum		
